# Can Axial-Based Nodal Size Criteria Be Used in Other Imaging Planes to Accurately Determine “Enlarged” Head and Neck Lymph Nodes?

**DOI:** 10.1155/2013/232968

**Published:** 2013-07-24

**Authors:** Eric S. Bartlett, Thomas D. Walters, Eugene Yu

**Affiliations:** ^1^Princess Margaret Cancer Centre, Joint Department of Medical Imaging, University of Toronto, 610 University Avenue, Room 3-956, Toronto, ON, Canada M5G 2M9; ^2^Department of Health Policy, Management and Evaluation, Faculty of Medicine, University of Toronto, 155 College Street, Suite 425, Toronto, ON, Canada M5T 3M6; ^3^Department of Paediatrics, The Hospital for Sick Children, University of Toronto, 555 University Avenue, Toronto, ON, Canada M5G 1X8

## Abstract

*Objective*. We evaluate if axial-based lymph node size criteria can be applied to coronal and sagittal planes. *Methods*. Fifty pretreatment computed tomographic (CT) neck exams were evaluated in patients with head and neck squamous cell carcinoma (SCCa) and neck lymphadenopathy. Axial-based size criteria were applied to all 3 imaging planes, measured, and classified as “enlarged” if equal to or exceeding size criteria. *Results*. 222 lymph nodes were “enlarged” in one imaging plane; however, 53.2% (118/222) of these were “enlarged” in all 3 planes. Classification concordance between axial versus coronal/sagittal planes was poor (kappa = −0.09 and −0.07, resp., *P* < 0.05). The McNemar test showed systematic misclassification when comparing axial versus coronal (*P* < 0.001) and axial versus sagittal (*P* < 0.001) planes. *Conclusion*. Classification of “enlarged” lymph nodes differs between axial versus coronal/sagittal imaging planes when axial-based nodal size criteria are applied independently to all three imaging planes, and exclusively used without other morphologic nodal data.

## 1. Introduction

Detection and classification of metastatic lymphadenopathy in patients with mucosal squamous cell carcinoma (SCCa) of the head and neck are based upon careful evaluation of known patterns of nodal metastasis, anatomic nodal level boundaries, and nodal morphology [1–5]. Within the untreated neck, identification of nodal boundaries is highly reproducible and accurate and allows for proper communication of findings to the clinical services [1]. The evaluation of nodal morphology is more complex and requires a judicious application of multiple guidelines defining nodal size, shape, and density/signal intensity [2–5]. Additionally, it is important to define the relationship of a lymph node to the adjacent soft tissues, to other lymph nodes and to the expected patterns of nodal drainage and metastatic spread within the neck [2–5]. Unfortunately, even a careful review of the neck by computed tomography (CT) or magnetic resonance imaging (MRI) may yield a false negative rate of 15–20% [4] in detection of metastatic lymph nodes and is not reliable to detect regional occult nodal metastasis [5].

Nodal size is one of the most important morphologic features to detect metastatic nodal disease from a mucosal-based SCCa within the head and neck. Measurement guidelines for lymph nodes in the head and neck can be controversial [4, 6]. In our institution, lymph node measurements are performed along the long axis of the lymph node within the axial plane, according to criteria defined by Som [2]. Defining a lymph node by its longest dimension is contrary to the short-axis description as is typical in chest and body imaging. However, the majority of lymph nodes in the head and neck are easily palpated by the clinician and described in the longest dimension. Our long-axis measures, therefore, allow for improved communication with the head and neck surgeons and radiation oncologists.

Lymph node measurement guidelines refer to measures obtained within the axial plane [1, 2, 4]. However, the long axis growth of a metastatic node may not correspond to the axial plane. The purpose of this study is to evaluate if the classification of lymph nodes, based upon size criteria alone, differs depending on the plane in which the lymph nodes are measured.

## 2. Materials and Methods

### 2.1. Patients/Subjects

Fifty pretreatment staging head and neck CTs were retrospectively reviewed by two neuroradiologists with an expertise in head and neck oncology. All patients were enrolled from a PACS archive (MERGE Healthcare, Fusion eFilm version 2.1.2, Chicago, IL, USA) according to the following criteria: pre-treatment exams of consecutive patients with pathologically proven mucosal-based SCCa within the head and neck and at least one clinically suspicious or pathologically proven metastatic lymph node within the neck. Patients were excluded from the study if the CT exam was not before therapy; if they had a history of prior head and neck malignancy; or if they had prior surgery, chemotherapy, or radiation therapy to the head and neck. The study was approved by our center's research ethics board (project identification number: 08-0651-CE). Informed consent was not required for inclusion into this study or for the evaluation of each patient's CT images and electronic medical records.

### 2.2. Materials/Image Acquisition

All head and neck CT exams were performed using a Toshiba Aquilion 64 CT (Toshiba Worldwide, Markham, ON, Canada). Images were acquired between the frontal sinuses and the aortic arch with 0.5 mm × 0.3 mm helical rotation and 220 display field of view, delivering an estimated radiation dose of 700 mgy·cm. A total of 105 ccs of Visipaque 320 IV contrast (GE Healthcare, United Kingdom) were power-injected at 1.5 cc/second through an antecubital fossa 20–22 gauge venous angiocatheter. Axial, coronal, and sagittal images were reconstructed every 2 mm using soft tissue and bone algorithms.

### 2.3. Image Analysis/Interpretation

The axial-based size criteria for metastatic lymphadenopathy were used to detect “enlarged” lymph nodes within the axial, coronal, and sagittal planes. A lymph node was considered “enlarged” when its measurement was equal to or larger than the threshold values published for the long axis size criteria (level Ib = 1.5 cm, jugulodigastric lymph node = 1.5 cm, retropharyngeal lymph node = 0.8 cm, and all other lymph nodes = 1.0 cm) [2]. 

**Figure 1:**
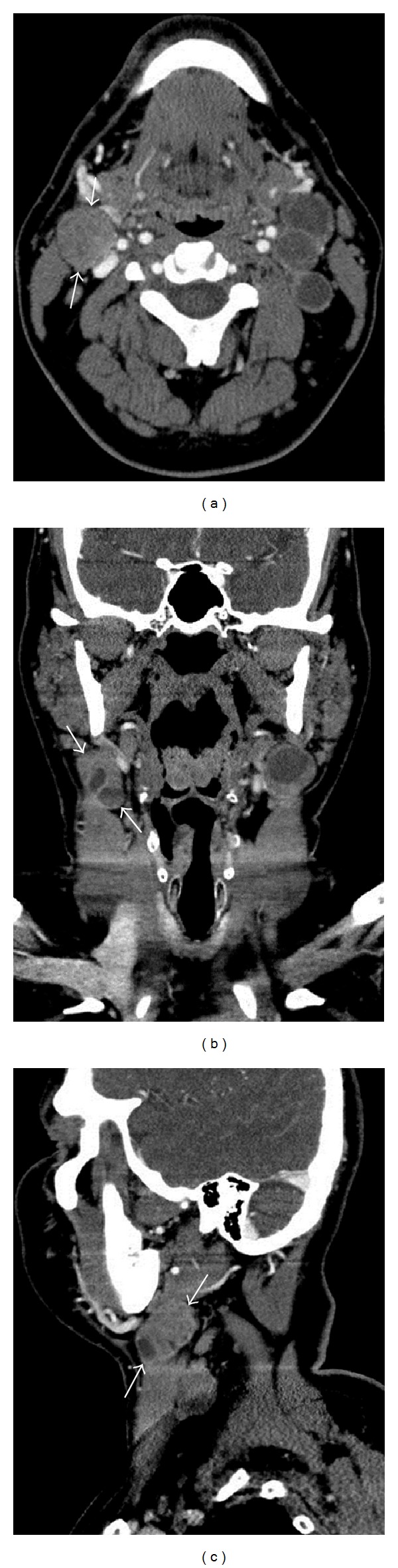
Detection of the largest lymph node in a patient with mucosal-based SCCa and neck lymphadenopathy by CT with IV contrast. Arrows demonstrate the longest axis of a selected “enlarged” lymph node within level IIA on (a) axial, (b) coronal, and (c) sagittal planes. The primary tumor was a left-sided oropharyngeal squamous cell carcinoma (SCCa). The selected lymph node shows partial central necrosis.

During the axial review, the largest lymph node meeting “enlarged” size criteria was selected within each nodal level and measured in its longest dimension (Figure 1(a)). The morphology of each selected node was characterized as either “normal,” “elongated,” or “round/suspicious.” “Normal” morphology included lymph nodes with a “lima” bean configuration, fatty hilum, well-defined smooth margins, and homogeneous density/signal intensity. “Elongated” nodes also had a well-defined smooth margin, homogeneous density/signal intensity, and fatty hilum but had a thin elongated configuration. The “round/suspicious” morphologic category referred to as lymph nodes without the benign “lima” bean configuration was missing a normal fatty hilum and/or had other abnormal morphologic features such as heterogeneous density/signal intensity due to cystic change, central necrosis, calcification, or abnormal contrast enhancement.

**Figure 2:**
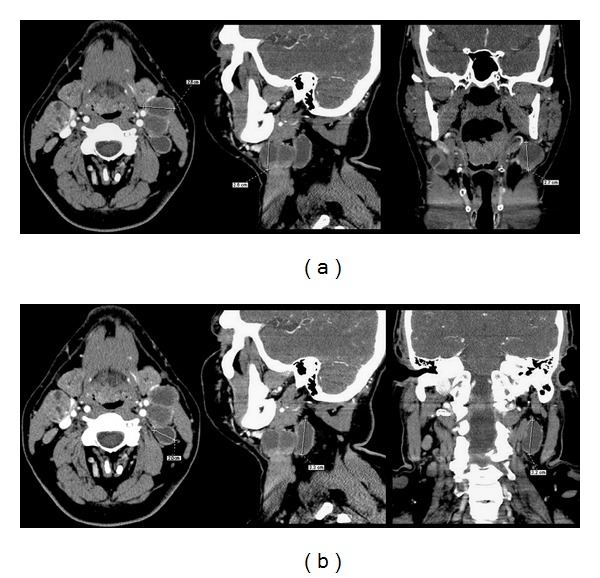
Selected “enlarged” lymph nodes that are not congruent between axial and coronal/sagittal reviews, allowing for two selected lymph nodes within a single ipsilateral nodal level. (a) The largest “enlarged” lymph node in the axial plane measured 2.8 cm, 2.6 cm coronal, and 2.7 cm sagittal (b) a different lymph node was the largest “enlarged” lymph node on the coronal (3.3 cm) and sagittal (3.2 cm) images with a smaller axial lymph node (2.0 cm) than selected in the original axial review. This is an oropharyngeal primary SCCa with partially necrotic/cystic lymph nodes.

The same methodology was adapted to the review of the coronal images, wherein the largest lymph node meeting “enlarged” size criteria was selected within each nodal level and measured in its longest dimension (Figure 1(b)). Each selected lymph node was cross-referenced to the axial images to determine if it corresponded to the “enlarged” lymph node selected in the axial review. If these selected lymph nodes did not correspond to each other, more than one “enlarged” lymph node was allowed per ipsilateral nodal level and measured in all three planes (Figure 2). The morphology of every selected lymph node was characterized as either “normal”, “elongated”, or “round/suspicious.”

Lastly, the sagittal images were reviewed for each head and neck, using identical methodology as in the axial and coronal reviews. Within the sagittal plane, the largest lymph node meeting “enlarged” size criteria was selected within each nodal level and measured in its longest dimension (Figure 1(c)). Again, each selected node was cross-referenced to the axial and coronal images to determine if the selected “enlarged” lymph node corresponded to the selected lymph nodes in the axial and coronal imaging planes.

All measures were performed independently. Any major differences between the two head and neck radiologists regarding lymph node selection or nodal level designation were reviewed in consensus conference.

### 2.4. Statistical Methods

All raw data were analyzed using the SPSS statistical software package for Windows (SPSS version 12.0.0, Chicago, IL, USA). Interobserver agreement was calculated using the Interclass Correlation Coefficient (ICC) for all lymph node measures. After analysis of the interobserver variability, the long axis measures were averaged between the two head and neck neuroradiologists and used in the final data analysis. Categorical data were created by classifying measures in all three planes as either “enlarged” or “normal,” according to the axial-based size criteria. The linear and categorical data for each plane were compared utilizing ICC, kappa, and McNemar's tests as appropriate with 95% confidence intervals.

## 3. Results

There were a total of 222 lymph nodes from our population of 50 patients that were considered “enlarged” in at least one plane. The median age at time of examination was 59 years old (IQR: 50.6–69.6 years). Seventy percent of the population was male (*n* = 35) and 69.8% of all selected lymph nodes were from male patients (*n* = 155). The majority of primary tumors were of the oral cavity and oropharynx (*n* = 13, *n* = 14, resp.). The remaining primary tumor sites included: larynx (*n* = 9), unknown primary (*n* = 6), retromolar trigone (*n* = 3), hypopharynx (*n* = 3), and nasopharynx (*n* = 2).

There were 11 instances where more than one lymph node was allowed in a single ipsilateral nodal level due to the longest maximum dimension occurring in different lymph nodes between the axial review and the coronal and sagittal reviews. Seven of these instances occurred in the level 2a nodal group, and 4 instances occurred in the level 3 nodal group. There were no instances where 3 different lymph nodes were identified within a single nodal level.

### 3.1. Size Criteria

**Figure 3:**
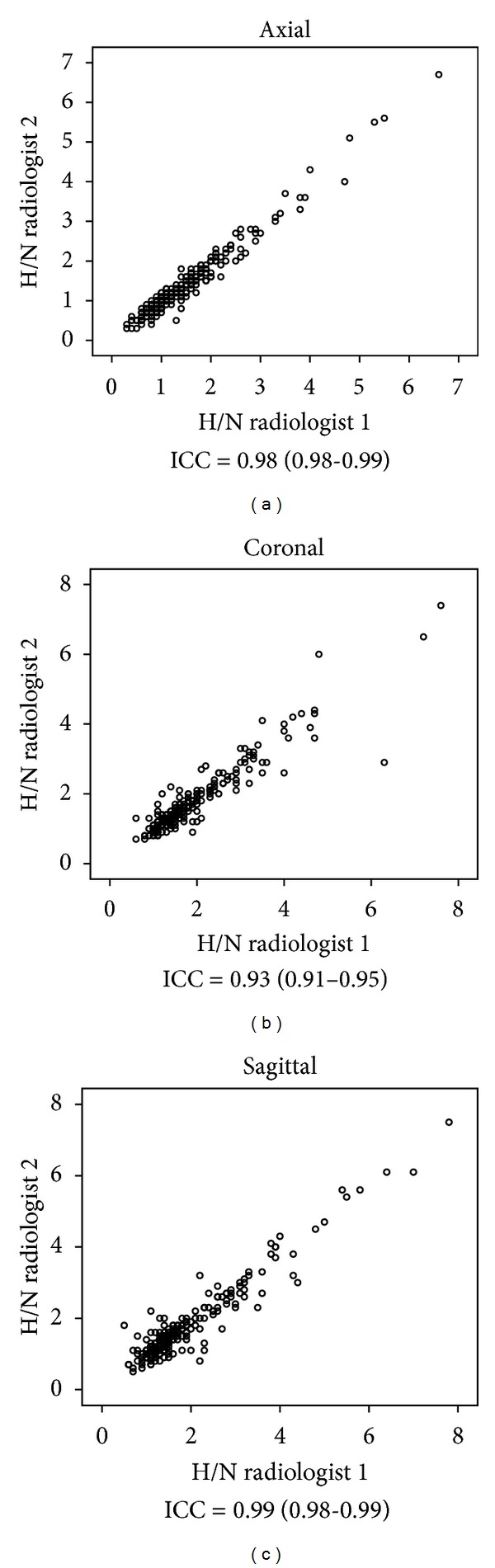
Interobserver agreement between the two head and neck (H/N) radiologists. “H/N radiologist 1” (E.Y.) has 7-year experience and “H/N radiologist 2” (E.S.B.) has 5-year experience in a dedicated head and neck oncology practice within a tertiary university hospital network.

The inter-observer agreement was excellent when comparing measures in each of the three respective planes (axial: ICC = 0.98 (0.98–0.99); coronal: ICC = 0.93 (0.91–0.95); and sagittal: ICC = 0.99 (0.98–0.99)) (Figure 3). The mean and median values were very similar for the measures in the coronal and sagittal planes (coronal: mean = 1.83 cm, median = 1.45 cm; sagittal: mean = 1.84 cm, median = 1.45 cm). The mean and median values for the measures in the axial plane were smaller than those in the coronal and sagittal planes (axial: mean = 1.46 cm, median = 1.2 cm).

**Figure 4:**
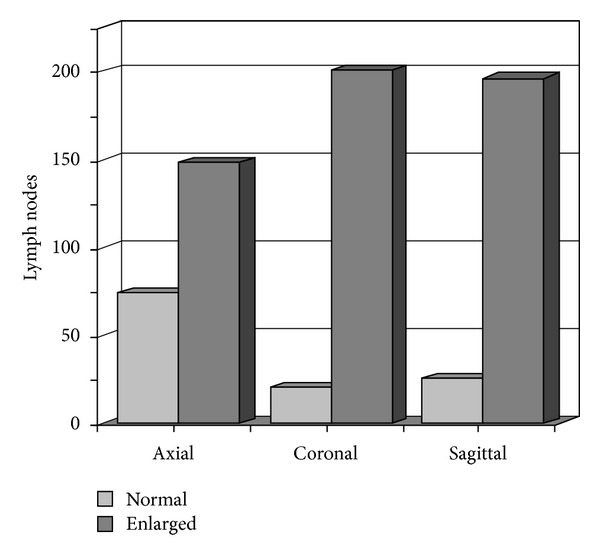
Comparison of “normal-” sized and “enlarged-” sized lymph node categories by imaging plane.

**Figure 5:**
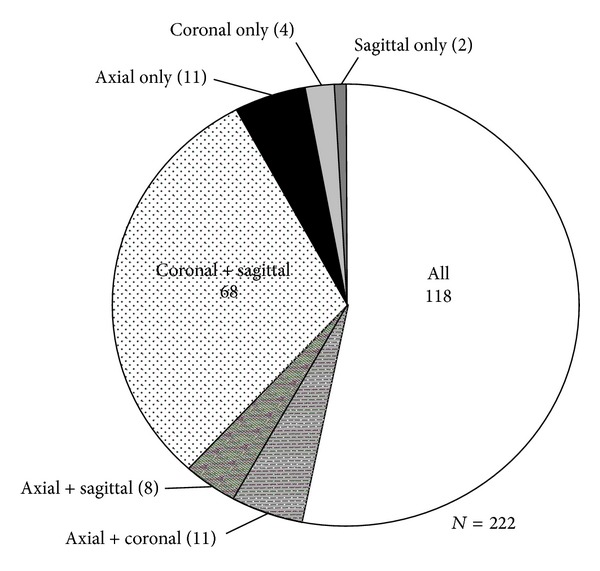
Distribution of “enlarged” lymph nodes by imaging plane.

By applying the axial-based longest dimension size criteria to define “enlarged” lymph nodes within all three planes, a total of 66.7% (*n* = 148) of the 222 measured nodes would be classified as “enlarged” if measured only within the axial plane. A total of 90.5% (*n* = 201) of all measured lymph nodes would be considered “enlarged” if the size criteria were applied only to the coronal measures. By applying the size criteria only to the sagittal plane, 88.3% (*n* = 196) of the measured lymph nodes would be considered “enlarged” (Figure 4). A total of 53.2% (*n* = 118) of all lymph nodes were classified as “enlarged” by all three planes. A total of 30.6% (*n* = 68) of lymph nodes were classified as “enlarged” within both the coronal and sagittal planes that were not enlarged by axial measures (Figure 5).

The ICC statistics show that the agreement between the axial versus coronal measures (ICC = 0.77, 0.56–0.87) and the axial versus sagittal measures (ICC = 0.77, 0.56–0.86) was not as good as the agreement between the coronal versus sagittal measures (ICC = 0.96, 0.94–0.97) (Table 1). 

The kappa statistics show that the level of agreement between categorical data (“enlarged” versus “normal” size) is also not as good for the axial versus coronal (*k* = − 0.09) and axial versus sagittal planes (*k* = − 0.07), in comparison to the level of agreement for the coronal versus sagittal (*k* = 0.44) categorical data. The McNemar statistic shows a systematic misclassification of “enlarged” lymph nodes when comparing axial versus the coronal planes (*P* < 0.001) and axial versus the sagittal planes (*P* < 0.001). The McNemar statistic shows good agreement in the classifications between the coronal and sagittal planes (*P* = 0.42) (Table 1). 

### 3.2. Nodal Morphology

Of the 148 lymph nodes classified as “enlarged” within the axial plane, a majority of these lymph nodes had a “round/suspicious” morphology (68.9%, *n* = 102). There were 18 (12.2%) of these 148 “enlarged” lymph nodes with an “elongated” morphology and 28 (18.9%) lymph nodes with a “normal” morphology. There were 74 (33.3%, *n* = 222) lymph nodes measured within the study that did not meet the size criteria within the axial plane. Only 4.1% (*n* = 3) of these “normal” sized axial nodes had a “round/suspicious” morphology. The remaining 71 “normal” sized axial nodes were either “elongated” (*n* = 44, 59.5%) or “normal” (*n* = 27, 36.5%) in their morphology (Table 2).

A total of 201 lymph nodes (90.5%, *n* = 222) were classified as “enlarged” within the coronal plane. Nearly half of these lymph nodes had a “round/suspicious” morphology (47.7%, 96/201). The percentage of “enlarged” lymph nodes that had an “elongated” morphology in the coronal plane (28.9%, 58/201) was over twice that of “enlarged” nodes in the axial plane (12.2%, 18/148). There were 21 (9.5%, *n* = 222) “enlarged” lymph nodes measured within the study that did not meet size criteria within the coronal plane. Over half (52.4%, *n* = 11) of the “normal” sized lymph nodes in the coronal plane had a “round/suspicious” morphology. Only 2 of the “normal” sized lymph nodes had an “elongated” shape, and only 8 had a “normal” shape (9.5% and 38.1%, resp.) (Table 2).

Within the sagittal plane, there were a total of 196 (88.3%, *n* = 222) lymph nodes that were classified as “enlarged.” Over half of these “enlarged” lymph nodes had a “round/suspicious” morphology (52.0%, 102/196). The percentage of “enlarged” lymph nodes that had an “elongated” morphology was 28.6% (56/196), nearly identical to the percentage within the coronal plane (28.9%, 58/201). There were a total of 26 (11.7%, *n* = 222) “enlarged” lymph nodes measured within the study that did not meet the size requirement within the sagittal plane. Only 19.2% (5/26) of the “normal” sized lymph nodes had a “round/suspicious” morphology, with a majority having a “normal” morphology (65.4%, 17/26). Only 4 (15.4%, *n* = 26) of the “normal” sized lymph nodes had an “elongated” morphology (Table 2).

If we only consider lymph nodes as “enlarged” if they meet size criteria in all three planes (*n* = 118), the percentage of “round/suspicious” lymph nodes increases to 77.9% (*n* = 92). Of the remaining 26 “enlarged” lymph nodes in this group, half were “elongated” (11.0%, *n* = 13) and half were “normal” (11.0%, *n* = 13) in morphology.

## 4. Discussion

Although the measurement of lymph nodes within the head and neck is routinely performed in the axial plane on CT imaging, no imaging study has been performed to assess the measurements within the coronal or sagittal imaging planes. Given our current ability to create isotropic reformats in the coronal and sagittal planes, it makes intuitive sense to measure each node in its longest dimension, regardless of the imaging plane.

Our data show that lymph nodes can be measured with very low inter-observer variation within all three planes. The measurement data show that the axial measures are slightly smaller than those in the coronal and sagittal planes, despite a relatively acceptable ICC score, suggesting that the majority of the lymph nodes measured were not spherical. The categorical data show that the axial-based size criteria systematically misclassify lymph nodes within coronal and sagittal planes in comparison to the axial plane.

Since lymph nodes are not judged in clinical practice to be “normal” or “abnormal” based upon size criteria alone, we gathered additional morphologic data for every lymph node measured. These additional morphologic categories were very general, with the “round/suspicious” category functioning as our target category for all suspicious morphologic features other than size.

### 4.1. Morphology in the “Enlarged” Category

When comparing the data based upon the morphologic categories, the highest percentage of “round/suspicious” lymph nodes within the “enlarged” category were within the axial plane. The percentage of “round/suspicious” lymph nodes that were classified as “enlarged” within the coronal and sagittal planes was smaller, accounting for approximately half of the detected “enlarged” lymph nodes in these planes.

The percentage of “elongated” lymph nodes within the coronal and sagittal “enlarged” category was nearly identical, and was greater than the percentage of “elongated” lymph nodes in the axial “enlarged” category. This suggests that the axial plane is less likely to incorrectly categorize a lymph node as “enlarged” when the node has an “elongated” configuration. Additionally, the greater numbers of “elongated” lymph nodes within the coronal and sagittal “enlarged” category help explain the difference in the overall numbers of “enlarged” lymph nodes in these planes in comparison to the axial plane.

### 4.2. Morphology in the “Normal-” Sized Category

There were only 3 “normal-” sized lymph nodes within the axial plane that had “round/suspicious” morphology. There were a higher number and percentage of lymph nodes with a “round/suspicious” morphology that were categorized as “normal” sized within the coronal and sagittal planes. Although the coronal and sagittal planes detected an overall higher number of “enlarged” lymph nodes in comparison to the axial plane, these higher percentages of “normal-” sized nodes with “round/suspicious” morphology indicate a greater risk of misclassifying lymph nodes in the coronal or sagittal planes if the size criteria are used alone without the benefit of other morphologic data.

There was a relatively low percentage of “normal”-sized lymph nodes with an “elongated” morphology in the coronal and sagittal planes in comparison to those in the axial plane. This further suggests that the axial plane is superior to the coronal and sagittal planes in correctly classifying the non-suspicious “elongated” lymph node as “normal.”

CT imaging of lymph nodes and the use of size criteria to evaluate head and neck cancer continue to be relevant, despite more advanced imaging techniques that can provide functional or physiologic nodal information. From the patient's perspective, CT imaging is better tolerated in patients with head and neck cancer. From a prespective of resource utilization, CT is relatively inexpensive, relatively accessible within most communities, and quick, has high reproducibility, and produces images with high anatomic precision. More advanced imaging techniques may eventually become more common; however, in the current era of austerity, access to such expensive technologies should be reserved for select difficult cases.

## 5. Conclusion

Classification of “enlarged” lymph nodes differs between the axial plane and the coronal and sagittal planes when nodal size criteria are applied independently to all three planes and exclusively used without the benefit of other morphologic nodal data. Modifications to the axial-based size criteria may increase our detection of metastatic lymph nodes; however, any adjustment of the size criteria will alter the sensitivity and specificity of any threshold values. A prospective study with pathologic confirmation of nodal metastasis should precede any attempt to modify axial-based size criteria or establish size criteria for the coronal or sagittal planes. The lack of pathologic confirmation of “enlarged” nodes is a clear limitation to this study. Although this study primarily evaluated nodal size, this was done specifically to evaluate differences between the imaging planes. Nodal size alone is not sufficient to properly evaluate metastatic neck lymphadenopathy in metastatic SCCa of the head and neck.

## Figures and Tables

**Table 1:** Evaluation of measurement and categorical data between imaging planes.

	ICC	Kappa	McNemar
Axial versus coronal	0.77 (0.56–0.87)	−0.09	*P* < 0.001
Axial versus sagittal	0.77 (0.56–0.86)	−0.07	*P* < 0.001
Coronal versus sagittal	0.96 (0.94–0.97)	0.44	*P* = 0.42

*P* < 0.05.

**Table 2:** Nodal morphology within the “enlarged-” and “normal-” sized categories by imaging plane.

	“Round/suspicious”	“Elongated” shape	“Normal” shape	*N*
Axial				
“Enlarged” size	68.9% (102/148)	12.2% (18/148)	18.9% (28/148)	66.7% (148/222)
“Normal” size	4.1% (3/74)	59.5% (44/74)	36.5% (27/74)	33.3% (74/222)
Total	**48.6% (105/222)**	**27.9% (62/222)**	**24.8% (55/222)**	**222**
Coronal				
“Enlarged” size	47.7% (96/201)	28.9% (58/201)	23.4% (47/201)	90.5% (201/222)
“Normal” size	52.4% (11/21)	9.5% (2/21)	38.1% (8/21)	9.5% (21/222)
Total	**48.2% (107/222)**	**27.0% (60/222)**	**24.8% (55/222)**	**222**
Sagittal				
“Enlarged” size	52.0% (102/196)	28.6% (56/196)	19.4% (38/196)	88.3% (196/222)
“Normal” size	19.2% (5/26)	15.4% (4/26)	65.4% (17/26)	11.7% (26/222)
Total	**48.2% (107/222)**	**27.0% (60/222)**	**24.8% (55/222)**	**222**
